# Use of the Theory of Planned Behaviour to assess factors influencing the identification of students at clinical high-risk for psychosis in 16+ Education

**DOI:** 10.1186/s12913-015-1074-y

**Published:** 2015-09-23

**Authors:** Debra A. Russo, Jan Stochl, Michelle Painter, Gillian F. Shelley, Peter B. Jones, Jesus Perez

**Affiliations:** CAMEO Early Intervention in Psychosis Service, Cambridgeshire and Peterborough NHS Foundation Trust, Block 7, Ida Darwin Site, Fulbourn Hospital, Fulbourn, Cambridge, CB21 5EE UK; Department of Psychiatry, University of Cambridge, Cambridge, UK; NIHR Collaboration for Leadership in Applied Health Research & Care, Cambridge, UK

**Keywords:** Early intervention, Psychosis, High risk, TPB questionnaire, Schools, Teachers, Intention

## Abstract

**Background:**

The longer psychotic disorders are untreated the worse their prognosis. Increasing the awareness of early psychosis by professionals who come into regular contact with young people is one strategy that could reduce treatment delay. As teachers engage with students on a daily basis, their role could be exploited to increase awareness of the early signs of psychosis. This study employed the Theory of Planned Behaviour (TPB) to identify and measure factors that influence identification of students at high-risk (HR) of developing psychosis in 16+ educational institutions.

**Methods:**

An elicitation phase revealed beliefs underlying teachers’ motivations to detect HR students and informed the construction of a preliminary 114-item questionnaire incorporating all constructs outlined in the TPB. To define the determinants of teachers’ *intention* to identify HR students, 75 teachers from secondary and further education institutions in 12 counties surrounding Cambridgeshire completed the questionnaire. A psychometric model of item response theory was used to identify redundant items and produce a reduced questionnaire that would be acceptable to teachers.

**Results:**

The final instrument comprised 73 items and showed acceptable reliability (*α* **=** 0.69-0.81) for all direct measures. Teacher’s confidence and control over identification of HR students was low. Although identification of HR students was considered worthwhile, teachers believed that their peers, students and particularly their managers might not approve. Path analysis revealed that direct measures of *attitude* and *PBC* significantly predicted *intention,* but *subjective norm* did not. *PBC* was the strongest predictor of *intention*. Collectively, the direct measures explained 37 % of the variance of *intention* to identify HR for psychosis.

**Conclusions:**

This research demonstrated how the TPB can be used to identify and measure factors that influence identification of students at HR of developing psychosis in 16+ educational institutions and confirmed the feasibility, reliability and acceptability of a TPB-based questionnaire for teachers. Consideration of the key determinants of identification in schools will facilitate the design of successful educational intervention strategies with the potential to reduce treatment delays for HR students.

## Background

The longer psychotic disorders are untreated the worse their prognosis [1–3], with some individuals remaining untreated for up to 2 years [4]. This finding has led to efforts to detect psychotic disorders early to minimise the developmental [5], social [6] and biological [7] deterioration that can occur after a prolonged duration of untreated psychosis (DUP). Increasing the awareness of signs and symptoms of early psychosis by professionals who come into regular contact with young people is one strategy that has been investigated to reduce treatment delay. Indeed, this approach has been recommended by the UK Department of Health [8]. Thus, if we could identify people who might be at clinical high-risk for psychosis (HR), we could also increase opportunities to reduce DUP. The importance of this is corroborated by the finding that the risk of developing psychosis is several hundred times higher in individuals that meet the high risk criteria when compared to the general population [9].

Initial psychotic symptoms typically have their onset and maximum impact in late adolescence and early adulthood [10]. It has been reported that adolescents (up to the age of 18) have a longer DUP than adults (over 18) [11]. Although it has been claimed that general practitioners (GPs) are most frequently the first contact when a young person is developing psychosis [12], it is of concern that other findings indicate adolescents rarely seek help from GPs concerning their emotional well-being and those with mental health problems do not visit the GP more frequently than those without them [13]. In light of the fact that teachers come into contact with students on a daily basis, sometimes for several hours at a time, their role could be exploited to increase awareness of the early signs of psychosis and speed up the referral of these potentially at risk students to Early Intervention Services (EIS).

Two intensive health promotion and information campaigns have included components that provided knowledge about early symptoms of psychosis to teachers [14, 15]. Both studies claimed to increase referrals to early detection teams and to significantly reduce DUP. However, the number of referrals specifically from the teachers was not reported. Mental health literacy training programmes for teachers have resulted in increased knowledge of the early signs of psychosis [16] and earlier, more appropriate referrals of pupils to mental health services [17, 18]. Indeed, a recent systematic review recommended the development of initiatives targeting non-health service professionals, such as teachers, to enhance help-seeking behaviour and therefore reduce service delays [19].

To date, only two studies have evaluated teacher’s knowledge concerning psychotic symptoms and how to access help for these individuals [20, 21]. Although teachers may be in a fundamental position to identify possible signs of psychosis and the majority are able to recognise these symptoms in their students, they are less likely to be aware of the mental health services available for young people or know how to access the appropriate help and services [20, 21]. The additional finding that teachers are willing to engage in further training in this area and cooperate with specialist teams to obtain support [20, 21] suggests an educational intervention to raise awareness and increase referrals of students at HR for psychosis in this group of professionals will be fruitful. However, unlike the more discernable symptoms of psychosis, identifying young people who might be in the prodromal phase of a psychotic illness is more challenging. The earliest signs of a psychotic disorder are multifaceted, non-specific and have common characteristics with the initial stages of other disorders [10]. This indicates the need for in-depth training to help teachers correctly identify the symptoms and those most at risk.

Before attempting to design an educational programme to help teachers detect students who may be at HR of developing psychosis, it is necessary to explore the factors that are currently influencing their identification. To our knowledge, no research to date has attempted to identify factors that influence the detection of students at risk of developing psychosis in schools. If it is assumed that teacher’s identification of HR students is a form of human behaviour, it can be described in terms of general theories relating to human behaviour. This provides a theoretical framework to help identify key determinants of that behaviour and propose options for its modification.

Interventions to change professional practice are often limited by the lack of an explicit theoretical and empirical basis [22]. The use of theory advances behavioural science [23] because it provides a generalisable framework for predicting and interpreting behaviour, informs the design of interventions and enables the evaluation of potential causal mechanisms [24].

### Theoretical framework

**Fig. 1:**
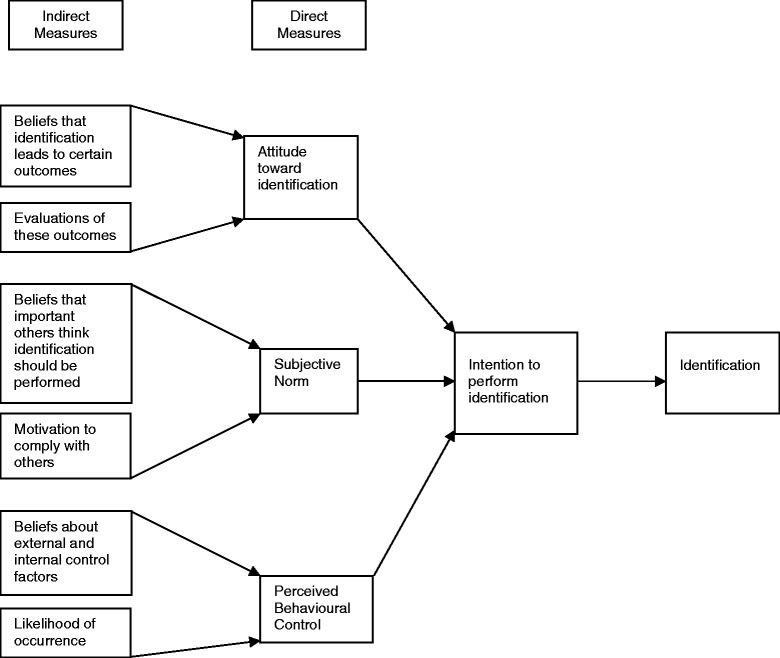
The Theory of Planned Behaviour

The Theory of Planned Behaviour [TPB; 25, 26] (Fig. 1) was selected because it provides clear definitions of constructs and is supported by a comprehensive body of correlational evidence [27]. The TPB provides a simple and efficient framework for use in the investigation of an individual’s intent to perform context-specific actions. The TPB assumes that the majority of human behaviour is goal-directed, socially influenced [25], and that individuals are logical and rational in their decision making [28]. It is a deliberative processing model that implies individuals make behavioural decisions based on careful consideration of available information [29]. In addition, it recognises the necessity of estimating the extent to which the individual is capable of exercising control over the behaviour in question [30]. The model’s ability to consider internal (e.g. abilities; knowledge) and external (e.g. opportunity; cooperation of others) control factors in relation to performing a behavior [31] is important in professional contexts such as educational institutions, where both factors may influence teacher’s behaviour.

The TPB proposes that the act of identifying students at HR for psychosis during the school day is predicted by the strength of a teacher’s *intention* to identify these students. This *intention* is guided by three considerations: the teacher’s personal evaluation of engaging in the identification of HR students (*attitude*); the intensity of social pressure from salient referents that the teacher perceives regarding the adoption of this behaviour (*subjective norm*) and the perceived ease or difficulty of identifying HR students based on both past experience and anticipated barriers (*perceived behavioural control*; *PBC*) [26].

The TPB has also been used to explain teachers’ intentions and behavior in the classroom [e.g.^32^, ^33^]. However, only one study has used the TPB to predict teachers’ intentions to refer students to mental health professionals [34]. Intentions were predicted by all the TPB variables; which accounted for 58 % of the variance associated with predicting their intention to refer.

This study describes the design and testing of items for a self-completion questionnaire to be used within 16+ educational institutions to identify and measure the factors that influence the identification of students at HR for psychosis using TPB. Results from this phase would inform the subsequent design of educational programs to evaluate the most effective way to help teachers identify these students.

## Method

We followed the guidelines outlined by the co-author of the TPB [35] and reviews of current standard practice for its application [29]. We were also guided by recommendations from other researchers in this field [36]. The behaviour under investigation was defined as “*identifying students at HR for psychosis during the school day”*.

### Phase 1: Questionnaire development

Observing the theory, the three predictors of intention were measured by two methods. Firstly, 'directly', by asking teachers to summarise their overall i) evaluative reaction to the identification of students at HR for psychosis (*attitudes*), ii) perceptions of whether important others would approve of or be likely to engage in the identification of students at HR (*subjective norms*) and iii) perception of having, or not having control over the identification of students at HR for psychosis (*PBC*). Secondly, ‘indirectly’, by asking teachers about their specific beliefs associated with forming *attitudes*, *subjective norms* and *PBC* related to the identification of students at HR. These indirect measures are presumed to determine the more global reactions of the direct measures [26].

### Development of ‘indirect’ measures

The objective of this phase was to elicit commonly held beliefs about identifying HR students from teachers. This enabled the development of questionnaire items based on these salient beliefs. Beliefs are central to the TPB; they provide the cognitive and affective foundations for *attitudes, subjective norms*, and *PBC* [35]. An accurate understanding of the specific beliefs associated with identifying students at HR for psychosis provides insight into why teachers may execute particular behaviours [35]. Therefore, this information can be important in the design of effective educational interventions.

### Procedure

Two teachers working outside of the study boundaries were recruited to complete an elicitation questionnaire to help identify salient beliefs underlying motivations to identify students at HR for psychosis during the school day. Each was emailed a series of 12 questions to determine:

1. Behavioural Beliefs: most frequently perceived advantages and disadvantages associated with identification
2. Normative Beliefs: most important people or groups who would disapprove or approve of identification
3. Control Beliefs: perceived barriers or facilitating factors associated with identification

To represent a variety of experiences within the final questionnaire the respondents were instructed to discuss these issues with colleagues so that the answers reflected both personal experience and also that of other teachers.

### Analysis

Two researchers independently analysed the content of the responses to identify the beliefs for each of the three predictor variables. These are are summarised below:

#### Behavioural beliefs

##### Positive

1. Facilitates help with their particular problems
2. Avoids friends and peers withdrawing due to negative interpretation of symptoms
3. Avoids staff misinterpreting student’s symptoms as lack of interest
4. Increases awareness and understanding
5. Avoids disruption to other students in class
6. Being able to help and counsel appropriately
7. Having a positive attitude to mental health
8. There are positive outcomes for those identified at risk

##### Negative

1. Students at HR should not be at school/college
2. I am wary of students with mental health problems
3. Labelling associated with identification
4. Identified students will be treated differently - stigma
5. Students wrongly diagnosed by a teacher
6. Lack of knowledge in how to cope with young people with psychosis
7. Negative preconceived ideas about how a student will react to identification
8. Belief that it is not their responsibility to identify someone at risk

##### Normative beliefs

1. Impact from student’s family has to be considered
2. Parents/carers may disapprove
3. Students may disapprove
4. Educational system does not encourage identification students at risk (e.g. LEA/school/college/staff union/departmental policies, government guidance)
5. Senior management teams at school/college are unwilling to accept that students at risk are an issue

### Control beliefs

#### Barriers

1. Difficulties in coping when dealing with students with psychosis.
2. Lack of understanding about early psychosis.
3. Fear of getting it wrong.
4. Fear of repercussions from student/family
5. Expressing concern is only effective if there is a school-wide procedure for investigating those concerns.
6. Not knowing where to go for advice and help if they suspect a student is suffering
7. Time restrictions
8. Feeling pressurised in their job
9. Not being able to recognise the at risk symptoms

#### Facilitators

1. Access to information, knowledge and resources
2. Having a designated member of staff to coordinate the care of students who may demonstrate signs of being at risk

Following this stage, a questionnaire item was constructed to assess the strength of each behavioural, normative and control belief. Additionally, a corresponding item was developed to assess the impact each belief might have on identifying HR student (Table 1). These indirect items and their format were then agreed by the entire research team, to ensure that each belief was represented in the questionnaire.

**Table 1:** Examples of questionnaire items assessing indirect Attitude, Subjective Norm and PBC

Belief Strength	*N* Items	Sample Item	Impact of Belief	*N* items	Sample Item
Attitude	11	If I were to identify students at risk of developing psychosis at school or college it would maintain their social functioning (e.g. support networks & relationships)	Outcome evaluation for each attitudinal belief	11	Maintaining social functioning of students is unimportant-important : *Extremely unimportant - extremely important*
Subjective Norm	5	The student’s family think I should identify a student at risk of developing psychosis at school or college	Motivation to comply with each group or individual	5	How much do you care what a student’s family think you should do? *Not at all – very much*
Perceived Behavioural Control	10	We have a school/college-wide procedure for identifying students at risk of developing psychosis	The power each control belief exerts	10	Having a school/college-wide procedure would make identifying students at risk of developing psychosis : *Less likely – more likely*

### Development of ‘direct’ measures

Direct measures are a summary estimate of a teacher’s global *attitude, subjective norm* and *PBC* towards identifying students at HR for psychosis; and predictors of *intention* to perform such identification [36]. *Intention* captures the motivational factors that influence behaviours [31] and signifies a teacher’s decision to exert effort to attempt identification [26].

### Procedure

According to the TPB guidelines, the direct measures were tailored to specific behaviours and samples [36]. This process should not be guided by an arbitrary selection of questions or adopted items from previous studies [35]. Therefore, appropriate items for the target population (teachers of 16+ students) and specific context (during the teaching day) were agreed by the research team to reflect each direct construct (Table 2).

**Table 2:** Examples of questionnaire items measuring direct Attitude, Subjective Norm, PBC and Intention

TPB Construct		*N* Items	Sample Item
Attitude		8	Identifying a student at risk of developing psychosis at school/college would be *Harmful/beneficial*
Subjective Norms		4	People whose views I value within my profession would disapprove of me identifying students at risk of developing psychosis: *Strongly agree – strongly disagree*
Perceived Behavioural Control	Self-Efficacy	3	Identifying students at risk of developing psychosis at school/college would be: *Difficult- easy*
Controllability		2	The decision to identify students at risk of developing psychosis at school/college is beyond my control: *Strongly agree – strongly disagree*
Intention	Intention	3	I am committed to identifying students at risk of developing psychosis at school/college: S*trongly agree – strongly disagree*
Self-prediction		1	I expect to identify students at risk of developing psychosis at school/college :S*trongly agree – strongly disagree*

### Phase 2: Questionnaire construction

A 114-item preliminary version of the questionnaire was constructed including indirect and direct measures for a*ttitude, subjective norm, PBC and intention*. The questionnaire included instructions regarding its completion and an introduction about how an individual at HR for psychosis might present in consultation. Feedback questions concerning ambiguity, content, missing factors and format guided any necessary subsequent refinements. Finally, socio-demographic questions were added to describe the sample.

### Phase 3: Questionnaire evaluation and refinement

The aim of this phase was to evaluate the acceptability and feasibility of administering the questionnaire within a representative sample of teaching staff in 16+ educational institutions, in addition to evaluating its reliability.

### Procedure

Questionnaires and information sheets were posted to 790 teachers working at secondary schools with a sixth form (N = 13), sixth form colleges (N = 2) and further education colleges (N = 4) across 12 counties in the UK between November 2009 and May 2010. These educational institutions were selected via a Google® search for ‘Secondary and Further Education institutions in the counties surrounding Cambridgeshire’. Selection criteria included 1) institutions with high quality prospectuses 2) websites that provided contact names for the various courses on offer. The information sheet outlined all ethical issues and contained sufficient information to allow teachers to decide whether they consented to take part in the study or not. A postal reminder was sent to non-respondents three weeks later.

Ethical approval was granted by the Cambridgeshire East Research Ethics Committee as part of the NIHR research programme RP-PG-0606-1335.

### Analysis

A psychometric evaluation of the questionnaire was conducted to confirm that information obtained using a reduced-item final tool would still provide a sound basis for decision making.

A modern approach, in the form of a psychometric item response model – the polytomous graded response model [37] was used to examine the validity of each item within direct and indirect measures and to inform decisions regarding the removal of items. The internal consistency of the direct measures of *attitude, subjective norm and PBC* was assessed using Cronbach’s Alpha coefficient on both the original and reduced-item questionnaires. An internal consistency criterion is inappropriate for the evaluation of reliability of indirect measures [35], because they are formative rather than reflective indicators of the underlying construct [38]. Alternatively, correlations between direct and indirect measures of the same construct were calculated to confirm the convergent validity of the indirect measures. Confirmatory factor analysis [39] was conducted on all measures to assess the relative importance of each item on the total construct; thus confirming the structural conformity of the final questionnaire with the TPB. The relationship between *intention* and the indirect and direct measures were investigated using path analysis, with “*intention*” specified as the dependent variable. Path analysis was used to reveal the degree of fit between the TPB and actual data, in addition to providing an estimation of multiple regression equations linking the TPB variables [40].

Data were analysed using the statistical software package NCSS Version 7.1 [41] for descriptive statistics; item analysis for the purpose of identifying redundant items for removal from the questionnaire was conducted using MULTILOG and confirmatory factor analysis and path analysis was performed with Mplus Version 6.1 [42].

## Results

### Descriptive statistics of the respondents

Seventy five (9.5 %) teachers returned questionnaires. The mean time taken to complete the questionnaire was reported as 20.1 (SD = 9.6) minutes. The mean age of participating teachers was 44.3 (SD = 10.9). More female teachers (N = 50; 67 %) than male teachers (N = 25; 33 %) completed and returned the questionnaire. The mean number of years teachers had been teaching was 13.7 (SD = 10.6). The majority of the sample (N = 62; 83 %) reported never attending any kind of mental health training during their careers. Teachers reported average class sizes of 17 (SD = 6.3) students and estimated that the mean number of students they taught with a mental health problem was 5 (SD = 7.6).

### Psychometric properties of the questionnaire

#### Validity

The polytomous graded response model [37] was used to study the validity of items within specific constructs. Also, distribution of responses for each item was assessed. This allowed the identification of items that required rewording, and those that were redundant because they added little information or offered similar response patterns. For the indirect measures, items were eliminated because of their ambiguity or similarity to other items. Final decisions on item exclusion were based on extensive discussions within the research team to avoid invalidation of the questionnaire due to exclusion of essential items that had emerged during the elicitation procedure. Forty-three items were excluded, resulting in a 73-item final questionnaire. Subsequent analyses were conducted on this reduced scale.

Pearson’s correlations between the indirect and direct measures of the corresponding construct indicate whether indirect measures are well constructed and adequately cover the breadth of the measured construct [43]. With the exception of *PBC,* each set of indirect beliefs was highly correlated with their direct predictor of *intentions*: behavioural beliefs with *attitudes* (r = 0.43; p < 0.001); normative beliefs with *subjective norms* (r = 0.61; p < 0.001); and control beliefs with *PBC* (r = 0.28; p < 0.023).

Factor analysis was used to assess the structural conformity of the final questionnaire with the TPB. The resulting standardized coefficients can be interpreted as correlations between the measured construct and corresponding item. Higher coefficients indicate higher factor validity. Therefore, these items are superior at discriminating between teachers with low and high levels of the corresponding latent construct.

Table 3 shows the items with the highest factor validity within direct and indirect measures. Only one item within (each) direct *subjective norms,* direct *PBC*, indirect *attitude* and two items within indirect *subjective norms* showed a factor validity lower than 0.5. However, indirect *PBC* was less coherent. All items within this construct showed low intercorrelations, in accordance with Azjen’s [35] premise that internal consistency is not a necessary feature of indirect measures. Therefore, the use of factor analysis is questionable for this construct. The factor validity is reported mainly for completeness and should be interpreted with caution.

**Table 3:** Items with the highest factor validity within indirect and direct measures

**Direct Measures**		**Item**	**Scoring**	**Factor Validity**
Attitude		If I were to identify students at risk of developing psychosis at school or college, it would be *Inappropriate/appropriate* (for my role)	+1 - +7	r = 0.76
Subjective Norm		It is not expected of me that I identify at risk of developing psychosis at school or college *Strongly Agree/Disagree*	+1 - +7	r = 0.90
Perceived Behavioural Control		I am confident that I could identify students at risk of developing psychosis at school or college if I wanted to *Strongly Agree/Disagree*	+1 - +7	r = 0.89
**Indirect Measures**		**Item**	**Scoring**	**Factor Validity**
Attitude Belief components:	Behavioural beliefs *X*	If I were to identify students at risk of developing psychosis at school or college it would increase awareness and understanding of mental health issues *Strongly Agree/Disagree*	+1 - +7	r = 0.90
	Outcome evaluation	Increasing awareness and understanding of mental health issues at school or college is Unimportant/Important	−3 - +3	
Subjective Norm Belief components:	Normative beliefs *X*	The senior management team within my school thinks I should identify students at risk of developing psychosis at school or college *Strongly Agree/Disagree*	−3 - +3	r = 0.97
	Motivation to comply	How much do you care what the senior management team within your school thinks you should do? *Not at all/Very much*	+1 - +7	
Perceived Behavioural Control Belief components:	Control beliefs *X*	I have knowledge of a student’s mental health history at school or college *Rarely/Frequently*	+1 - +7	r = 0.76
	Influence of control	Knowledge of my student’s mental health history would make identifying a identify student at risk of developing psychosis at school or college *Difficult/Easier*	−3 - +3	

#### Reliability

The lower bound estimates of reliability assessed by Cronbach’s alpha for the original and reduced questionnaires are shown in Table 4. An acceptable level of *α* was set at >0.60 [35]. The values confirmed improvement for *subjective norms* and *PBC* in the reduced version. However, measurement precision for *intention* and *attitude* was slightly reduced but still large enough to be interpreted as acceptable. As a greater number of items in the questionnaire can artificially inflate the value of alpha [44] it was decided that a shorter questionnaire would be more acceptable to teachers and therefore a slightly reduced alpha was an acceptable compromise.

**Table 4:** Cronbach’s alphas for the direct measures of the original and reduced form questionnaires

Direct Measures	Original Questionnaire	Reduced Questionnaire
	114 items	73 items
Intention	0.82 (2.56)	0.81 (2.63)
Attitude	0.82 (3.67)	0.74 (3.86)
Subjective Norms	0.62 (3.45)	0.69 (2.77)
PBC	0.66 (3.54)	0.75 (2.91)

### Distribution of teachers’ scores for all TPB constructs

For the direct measures, including *intention*, the mean of the item scores was calculated to provide an overall construct score (See Table 3 for item scoring ranges). Beliefs are structured according to an expectancy-value framework. Individuals hold expectancies about the outcomes they anticipate if they behave in a particular way. Simultaneously, they also hold beliefs about the value of that outcome [26]. Therefore, indirect measures are calculated by multiplying individual belief components and then summing the products. For example, indirect *attitude* is calculated by multiplying the perceived likelihood of a particular outcome of the behaviour (behavioural belief strength) by the evaluation of that outcome (outcome evaluation) The resulting products are summed across all beliefs to create an overall attitude score (See Table 3 for the corresponding *subjective norm* and *PBC* belief components)

Table 5 summarises data obtained from the questionnaires. Higher scores indicate that a teacher intends to, is in favour of, experiences social pressure to, and feels in control of identifying students who may be at HR for psychosis.

**Table 5:** Descriptive statistics of Teachers’ responses for the indirect and direct measures

**Indirect Measures**	**Final no. of items**	**Mean**	**Standard Deviation**	**Standard Error**	**Minimum Score**	**Maximum Score**	**Possible range of total scores**
Attitude	22	123.9	51.1	6.11	−5	225	- 231 to + 231
Subjective Norm	10	3.1	28.0	3.26	−65	65	- 105 to + 105
PBC	20	29.7	27.1	3.13	−64	95	- 210 to + 210
**Direct Measures**	**Final no. of items**	**Mean**	**Standard Deviation**	**Standard Error**	**Minimum Score**	**Maximum Score**	**Mid-Scale Score**
Intention	4	16.5	6.0	0.70	4	28	16
Attitude	8	39.9	7.6	0.88	8	56	32
Subjective Norm	4	14.7	5.0	0.58	4	28	16
PBC	5	18.5	5.8	0.67	5	35	20

For indirect measures, mean scores reflected overall weakly positive attitudes towards identification, almost no favourable pressure to perform identification and very low control over the identification of students at HR for psychosis. *Subjective norm* was the lowest (3.1), which indicates a very weak level of positive control. *Attitude* was the highest, but still a low score (123.9).

Mean scores for direct measures were just above the mid-scale score for *intention* and *attitude*, and just below the mid-scale score for *subjective norm* and *PBC*. This suggests that teachers considered identifying students at HR for psychosis a worthwhile behaviour and would attempt identification during the school day. However, they believed that their peers or superiors might not approve this. Moreover, their confidence and control over identification was low.

### Prediction of ‘intention’

Path analysis revealed that only direct measures of *attitude* and *PBC* significantly predicted *intention. Subjective norm* did not predict *intention. PBC* was the strongest predictor of *intention* (regression coeff. = 0.46, p < 0.01), followed by *attitude* (0.39, p < 0.01).

Collectively, the direct measures explained 37 % of the variance of *intention* to identify HR for psychosis.

## Discussion

The purpose of this research was to design a questionnaire to expose and measure factors that might contribute to a teacher’s decision to attempt identification of students that may be at clinical HR of developing psychosis. This study was conducted because there was no evidence in the literature of the influence of the motivations and barriers teachers experience when making this decision.

Results from the analysis of the indirect measures revealed that increasing awareness and understanding of mental health issues was a source of personal positive beliefs, as demonstrated by the item with the highest factor validity within the indirect *attitude* construct. Notably, all normative beliefs were negative*.* The perception was that students, student’s family, professional colleagues and the educational system would not encourage the identification of students at HR for psychosis. The item with the highest factor validity indicated that a key source of social pressure came from the senior management team within school. The proposal that control beliefs should comprise separate measurement of controllability and self-efficacy [45] was supported by our study. However, control factors were the primary influence for control beliefs. The item with the highest factor validity indicated that knowledge of the student’s personal and family mental health history was an important facilitator of *PBC*. Facilitators of self-efficacy included access to support and the provision of a designated member of staff to co-ordinate the care of students. Lack of understanding and knowledge were the main barriers to self-efficacy. These findings replicated previous work revealing factors that might prevent teachers’ identifying psychotic symptoms in students [20, 21], thus demonstrating the validity of our questionnaire.

These results suggest a reoccurring theme of beliefs underlying a teacher’s decision to attempt identification of a student that might be at HR of developing psychosis: lack of access to information, knowlege and resources will all hinder teacher’s ientification behaviour. Identifying these particular beliefs reveals why teachers hold certain attitudes, subjective norms, and perceptions of behavioral control in relation to identifying students at risk of developing psychosis. This demostrates the value of using the TPB for designing effective programs to change teacher’s behavior. Equipped with this information, effective strategies can be designed to target these facilitators and barriers towards identification of at risk students.

Results from the analysis of the direct measures revealed that most teachers had positive *intentions* and *attitudes* towards identifying students at HR for psychosis. However, mean scores for each direct construct were around the mid-scale score, indicating scope for modification and improvement. The mean *PBC* score indicated a degree of negativity about control, suggesting that identifying students at HR was somewhat difficult for teachers, both in terms of self-efficacy and perceived control concerns. *Intentions* to identify HR students were most strongly predicted by *PBC.* This implies teachers’ perceptions of how confident they are that they are capable of identification and how much control they have over identification, are prominent motivational factors. This influence of *PBC* was also found in Lee’s work [34] with teachers and is consistent with previous research that reports teachers with lower self-efficacy referred fewer students to a student support team [46]. Accordingly, effective interventions would need to prioritise the development of strategies that targeted this potential causal mechanism to prompt behavioural changes in this population.

Our questionnaire proved to be reliable, with the analysis supporting the predictive power of the TPB with regards to *intention.* The combination of *attitude, subjective norm* and *PBC* explained 37 % of the variance of *intention* to identify students at HR for psychosis. This is almost equivalent to the average percentage (39 %) of explained variance in *intention* reported for a variety of behaviors in the latest meta-analytic review of the TPB [31]. Interestingly, *subjective norm* was the only direct measure not to perdict intention, supporting previous studies that proposed that *subjective norm* was the weakest explanatory variable of intention [31].

It appears the control factors identified in the elicitation exercise did not capture adequately all the important considerations related to *PBC*. We suspect that the low correlation between direct and indirect *PBC* was due to the lack of reliability for indirect *PBC*. There are several possible explanations for this result. Firstly, items within indirect measures are not expected to correlate strongly with each other as they reflect a dynamic latent construct [35]. Secondly, teachers’ *PBC* beliefs toward identifying students at HR of developing psychosis could be ambivalent if they believe that it is likely to produce positive as well as negative outcomes [35]. Thirdly, the *PBC* construct generated beliefs with the greatest diversity, therefore, fewer items were removed during validity analysis, with the aim of retaining important beliefs that influence teachers' perceptions of control. However, to create a questionnaire of acceptable length, valid items might have been excluded. Finally, the way in which teachers conceptualise the notion of control and difficulty [47] could have contributed to the discrepancy. For example, teachers may believe that identification of students at HR for psychosis is under their control, whilst also considering identification difficult to carry out. Consequently, the inclusion of numerous different aspects of control within the constructs could be a major contributory factor to the low correlation.

These propositions imply that the identification of HR students is intrinsically challenging for teachers, especially when considering identification is not straightforward for clinicians and researchers in the field with specialist training. However, if interventions to educate teachers focus on the providing skills and strategies for the identification of symptoms rather than an actual ‘diagnosis’ of HR, referral of HR students could be achieved. Additionally, the use of a dedicated liaison practitioner to provide ongoing support and augment training with this potentially challenging task would be advantageous.

The major strengths of this study were the thorough psychometric evaluation of our TPB questionnaire and the explicit theoretical framework. Since the majority of TPB questionnaires are used only once with a specific population and behaviour, a thorough psychometric evaluation is usually considered non-feasible and therefore omitted [48]. Our research provides an empirically-supported theoretical basis for the design of interventions in 16+ educational institutions to improve the identification of students at HR for psychosis.

Despite strenuous efforts, the response rate to our questionnaire was poor. The low response rate (9.5 %) from the invited sample (N = 790) was the most important limitation of the study, and potential risk of bias for the findings. External validity could have been undermined if respondents differed systematically from non-respondents, e.g. more positive attitude towards identification. However, the respondents were from a variety of locations with varying years of teaching experience, in a diverse selection of subjects and positions which arguably provides a representative sample of the target population and increases the generalisability of the results. It was not possible to conduct a detailed analysis of non-responses as the necessary sociodemographic information was not accessible. Nevertheless, future work should aim to increase the response rate.

It is not possible to fully understand why teachers chose not to respond to the questionnaire. However, research has revealed strategies that may help future studies to increase their sample. The majority of strategies outlined in a recent review [49] of methods to influence responses to postal questionnaires were applied in the present study. However, sending questions by recorded delivery, providing non-respondents with a second copy of the questionnaire and contacting participants before sending questionnaires all increased response rates and should also be considered by future research. Furthermore, the most effective strategy, if funds allow, is a monetary incentive, as response rates can be more than doubled when payment is offered [49]. Nevertheless, questionnaire length would remain the limiting factor as the TPB requires the inclusion of many items if it is to be used effectively to provide important insights into the issues that could be targeted to motivate behaviour change. The questionnaire in the present study was five pages long and it has been suggested that the optimum length is four pages [49]; hence efforts should be made to achieve this without compromising the theoretical content.

Our findings may have been limited by the use of self-reports as measures of beliefs and intention. As a result, the respondents might have unintentionally (‘social desirability’) or intentionally (‘faking good’) [50] expressed themselves more positively toward the identifying at risk students than they really were. However, inclusion of further questions in the questionnaire to assess this was not feasible. The questionnaires were already long enough to discourage some teachers from responding. Also, previous studies suggested that social desirability had a minimal impact on TPB models [51]. Moreover, returned questionnaires were anonymous, with no incrimination or benefits from participating. Also, current behaviours were not measured. Future research should not only rely on self-reports but include objective measures of behaviour.

## Conclusions

This research demonstrated how the Theory of Planned Behaviour can be used to identify and measure factors that influence identification of students at HR of developing psychosis in 16+ educational institutions. We have confirmed the feasibility, reliability and acceptability of a TPB-based questionnaire to identify teachers’ beliefs and intentions concerning the identification of students at clinical HR for psychosis. Detection of the key determinants of identification will suggest avenues for modification and facilitate the design of successful educational intervention strategies.

The questionnaire is available from the authors.

## Abbreviations

DUP: Duration of untreated psychosis
EIS: Early intervention services
GP: General practitioner
HR: Clinical high-risk for psychosis
PBC: Perceived behavioural control
TPB: Theory of planned behaviour
